# Supplementary material for: Hybrid CNN and multi-head attention model for analyzing epigenetic mechanisms and gene expression across fungal phylogenetic distances
Source: iScience. 2026 Aug 22;29(9):117280. doi: 10.1016/j.isci.2026.117280 (PMC13506266; doi:10.1016/j.isci.2026.117280)
Supplement: Supplementary file 1 [file mmc1.pdf]

iScience, Volume *29*

Supplemental information

Hybrid CNN and multi-head attention model for analyzing epigenetic mechanisms and gene expression across fungal phylogenetic distances

Laura Weinstock, Jenna Schambach, Anna Lara, Cameron Kunstadt, Ethan Lee, Elizabeth Koning, William Morrell, Wittney Mays, Warren Davis, and Raga Krishnakumar

Document S1

Supplementary Figure 1. Intra-species shallow regressor model battery performance using *N. crassa* on mean absolute error and regression AUROC metrics (related to Figure 3).

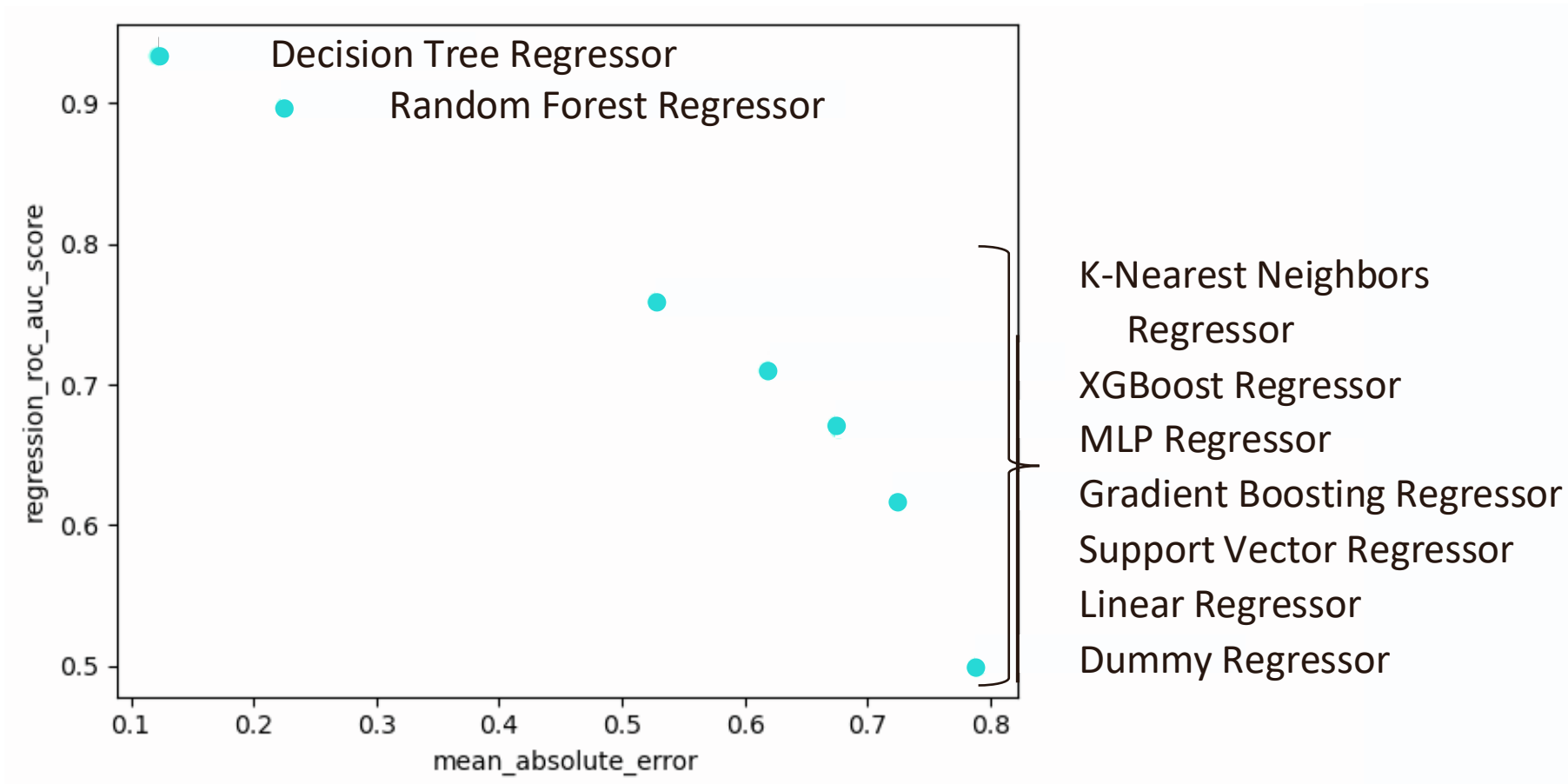

Supplementary Figure 2. Confusion matrices and metrics output table show K-nearest neighbors classifier performance across (A) 2, (B) 3, (C) 4, (D) 5 output bins for intra-species predictions using *N. crassa* data. Bin thresholds set to achieve equal # genes/bin. Colorbar indicates number of genes predicted for each cell (related to Figure 3).

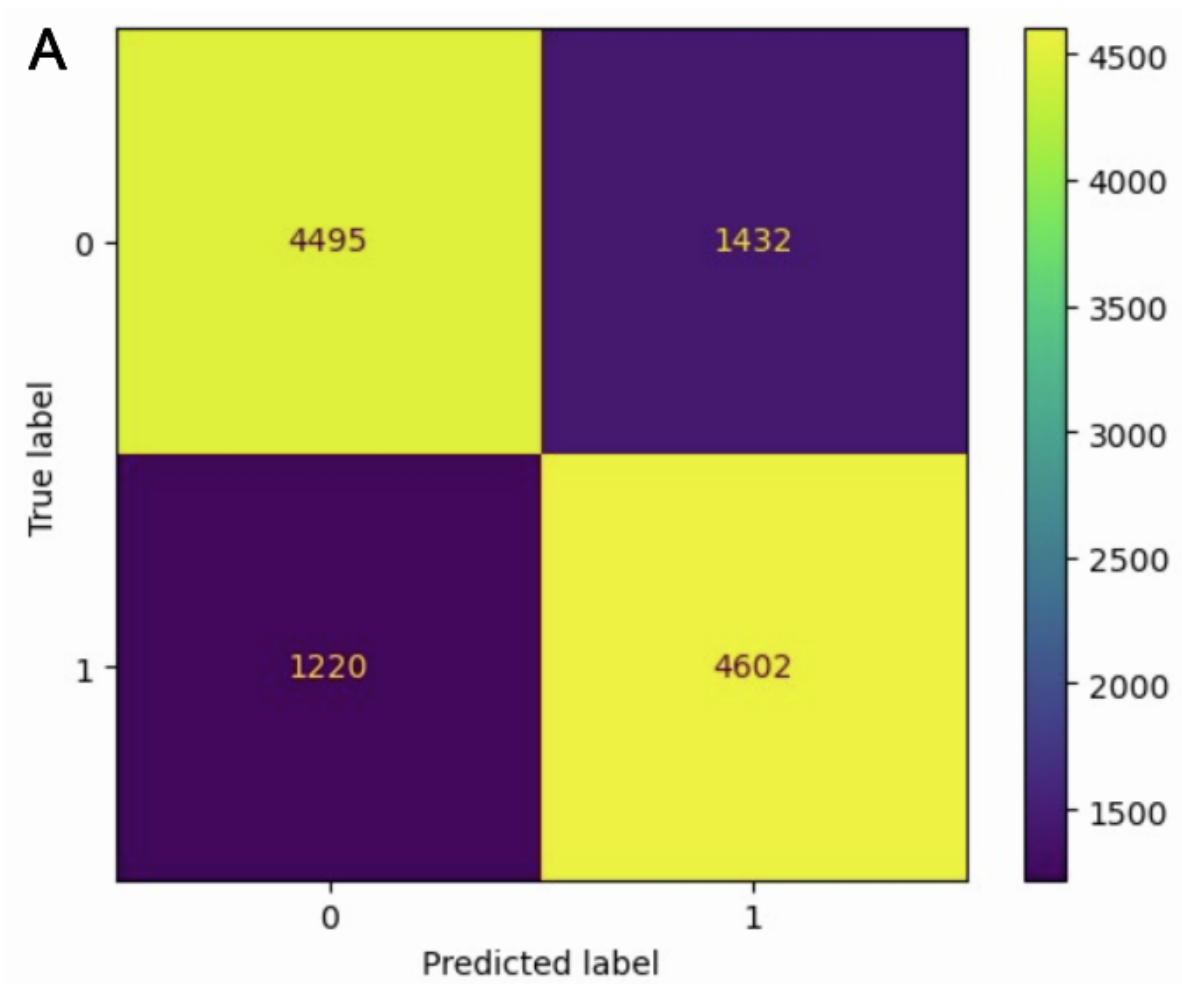

	precision	recall	f1-score	support
0	0.79	0.76	0.77	5927
1	0.76	0.79	0.78	5822
accuracy			0.77	11749
macro avg	0.77	0.77	0.77	11749
weighted avg	0.77	0.77	0.77	11749

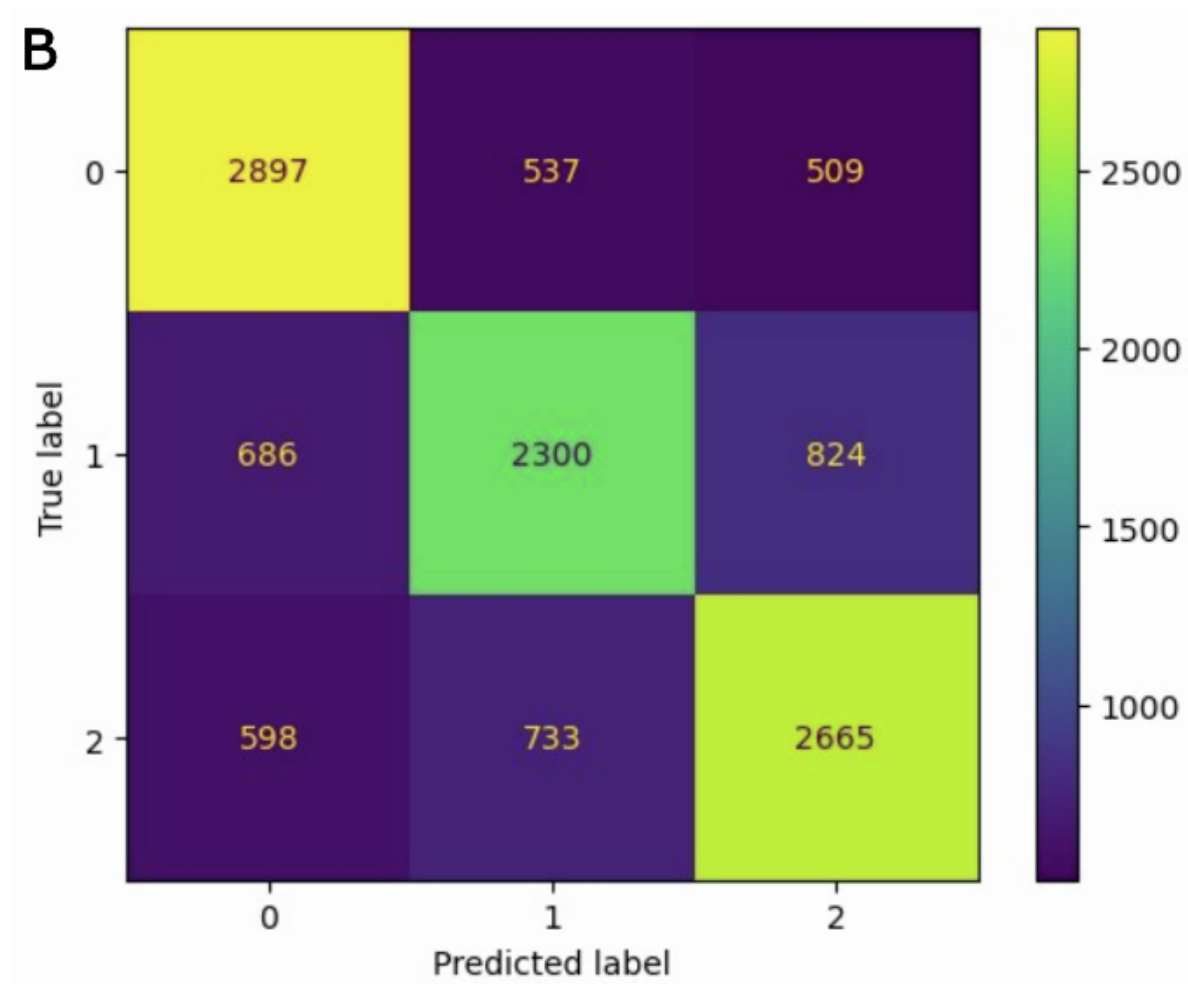

	precision	recall	f1-score	support
0	0.69	0.73	0.71	3943
1	0.64	0.60	0.62	3810
2	0.67	0.67	0.67	3996
accuracy			0.67	11749
macro avg	0.67	0.67	0.67	11749
weighted avg	0.67	0.67	0.67	11749

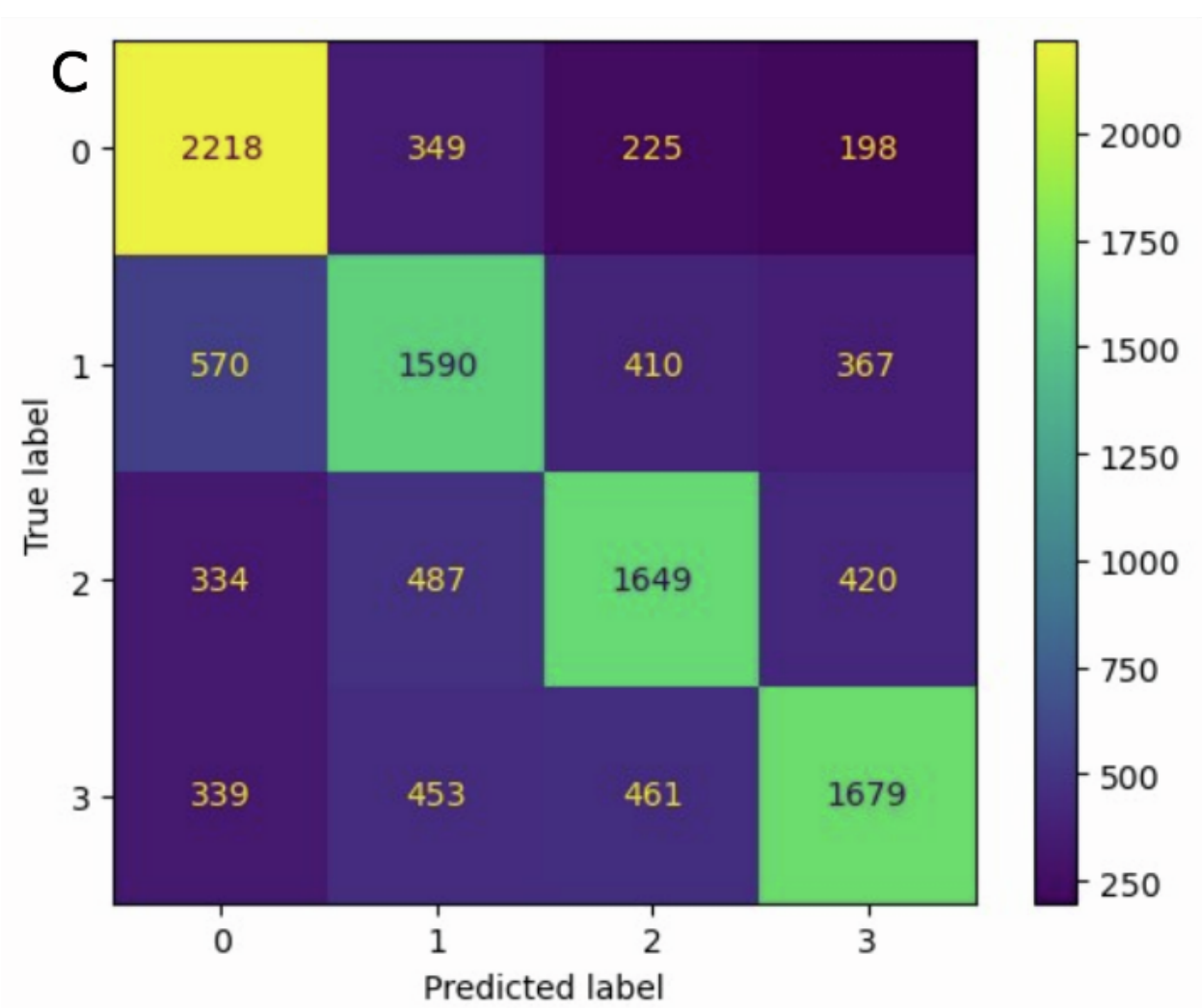

	precision	recall	f1-score	support
0	0.64	0.74	0.69	2990
1	0.55	0.54	0.55	2937
2	0.60	0.57	0.59	2890
3	0.63	0.57	0.60	2932
accuracy			0.61	11749
macro avg	0.61	0.61	0.60	11749
weighted avg	0.61	0.61	0.61	11749

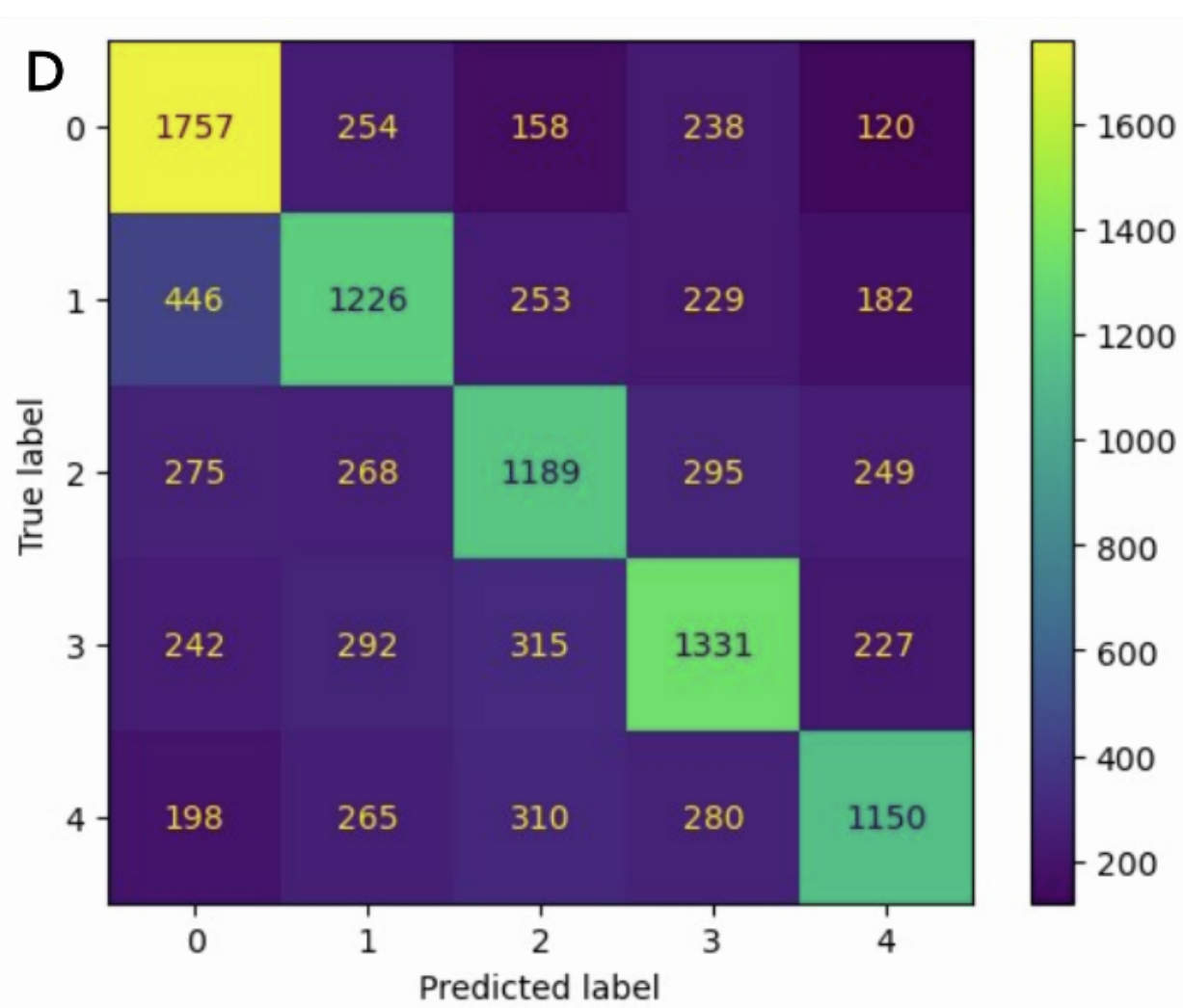

	precision	recall	f1-score	support
0	0.60	0.70	0.65	2527
1	0.53	0.52	0.53	2336
2	0.53	0.52	0.53	2276
3	0.56	0.55	0.56	2407
4	0.60	0.52	0.56	2203
accuracy			0.57	11749
macro avg	0.57	0.56	0.56	11749
weighted avg	0.57	0.57	0.56	11749

Supplementary Figure 3. Detailed shallow classifier model prediction performance results for each combination of species, model, TSS window, signal vs averaged features, and class output number for both inter- and intra- species prediction. Colorbar shows performance metric (accuracy, precision, or AUROC) score (related to Figures 3 and 4).

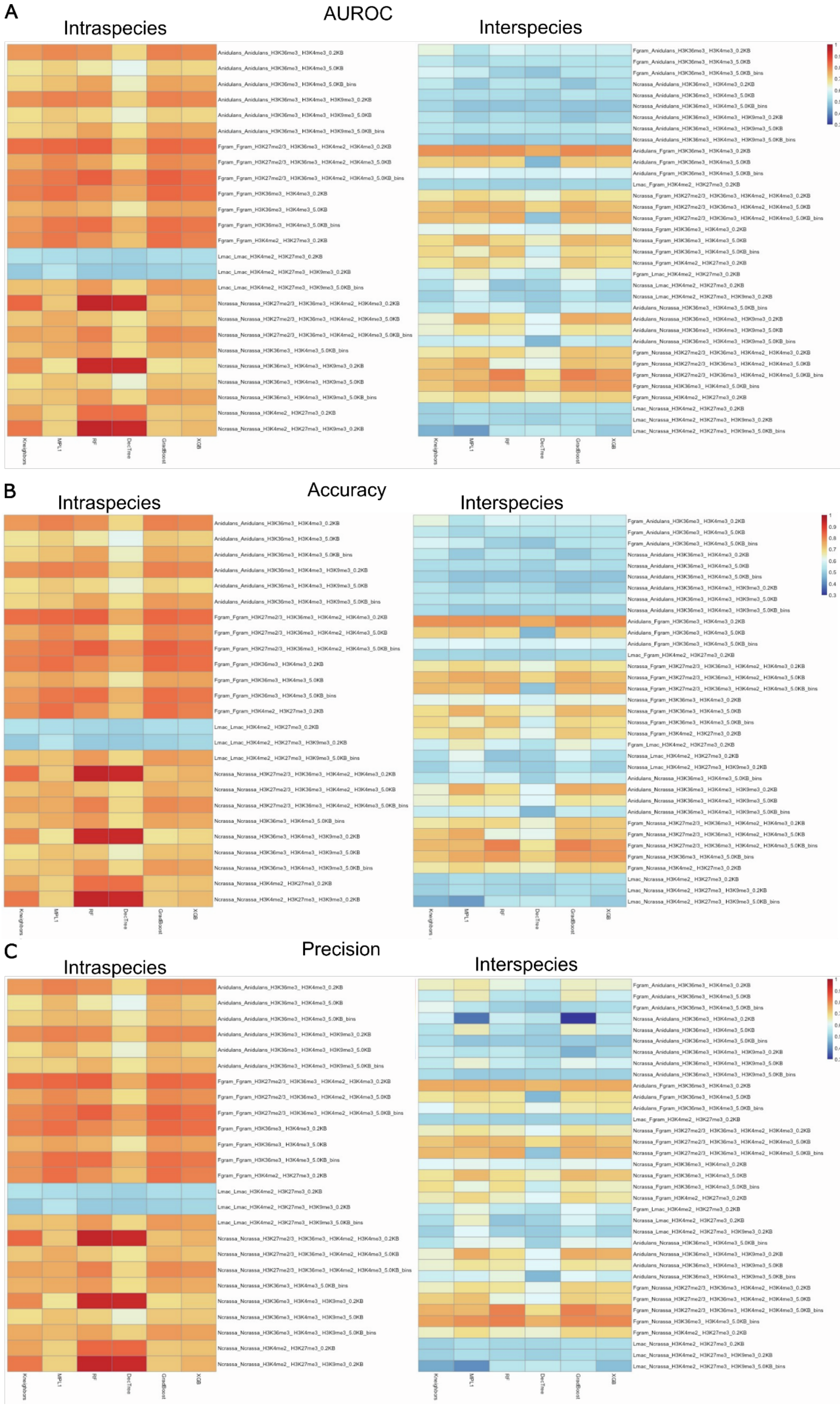
A
AUROC
Intraspecies
Interspecies
B
Accuracy
Intraspecies
Interspecies
C
Precision
Intraspecies
Interspecies

Supplementary Figure 4. Average shallow learning classifier performance for each number of overlapping modifications available for use in test for intra- (left) and inter- (right) species predictions. Boxes represent median +/- quartile for minimally n = 36 replicates **(**related to Figures 3 and 4).

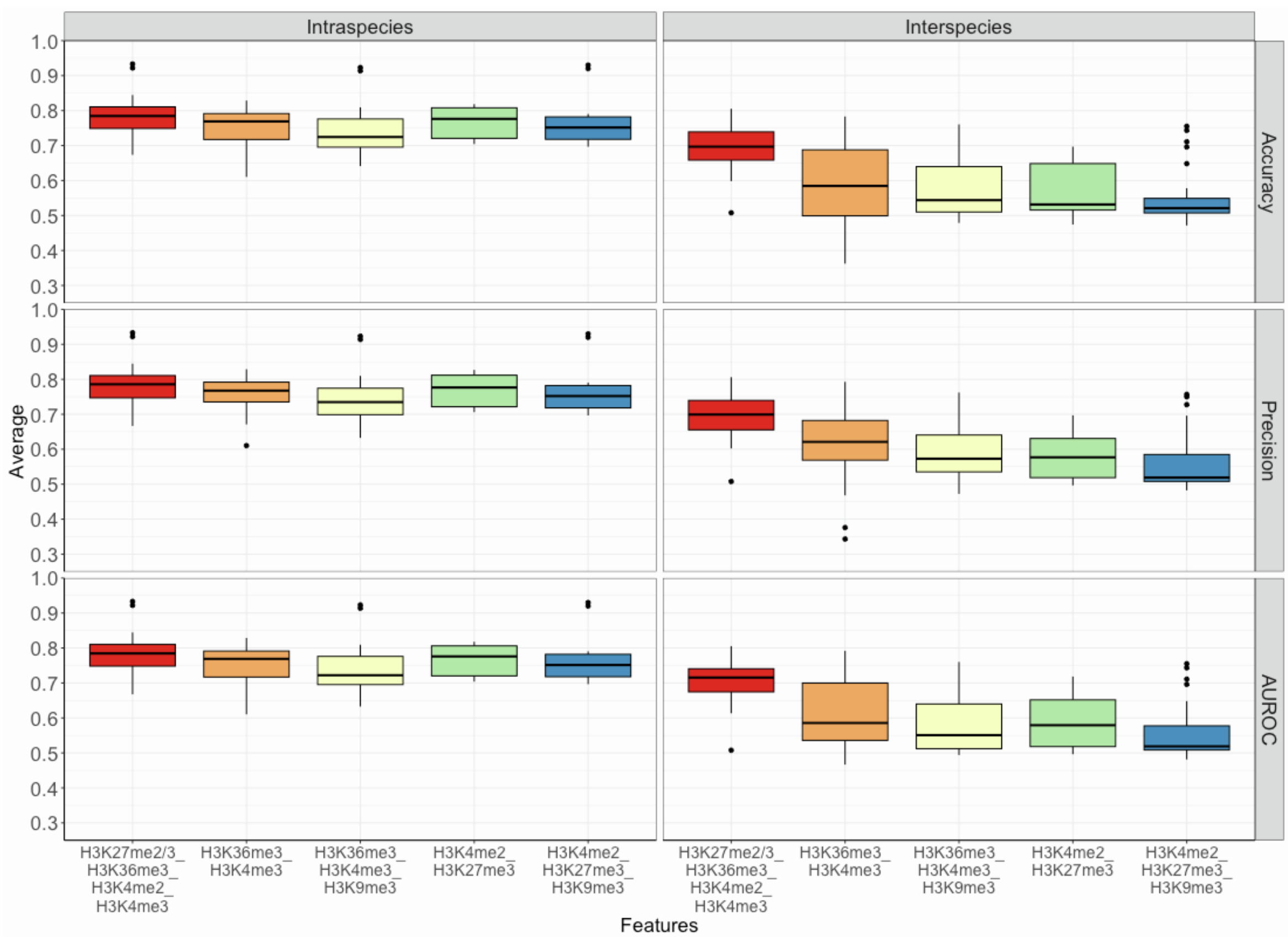

Supplementary Figure 5. Feature design impact on prediction performance. Average shallow learning classifier performance for intra- (left) and inter- (right) species predictions using varying feature input approaches: TSS window of 200bp or 5kb and epigenetic ChIPseq signal averaged over full TSS window or in 100bp bins returning a modified signal profile. Boxes represent median +/- quartile for n= 162, 108, 144 replicates for 200 bp average, 5000 bp average, and 5000 bp profile, respectively (related to Figures 3 and 4).

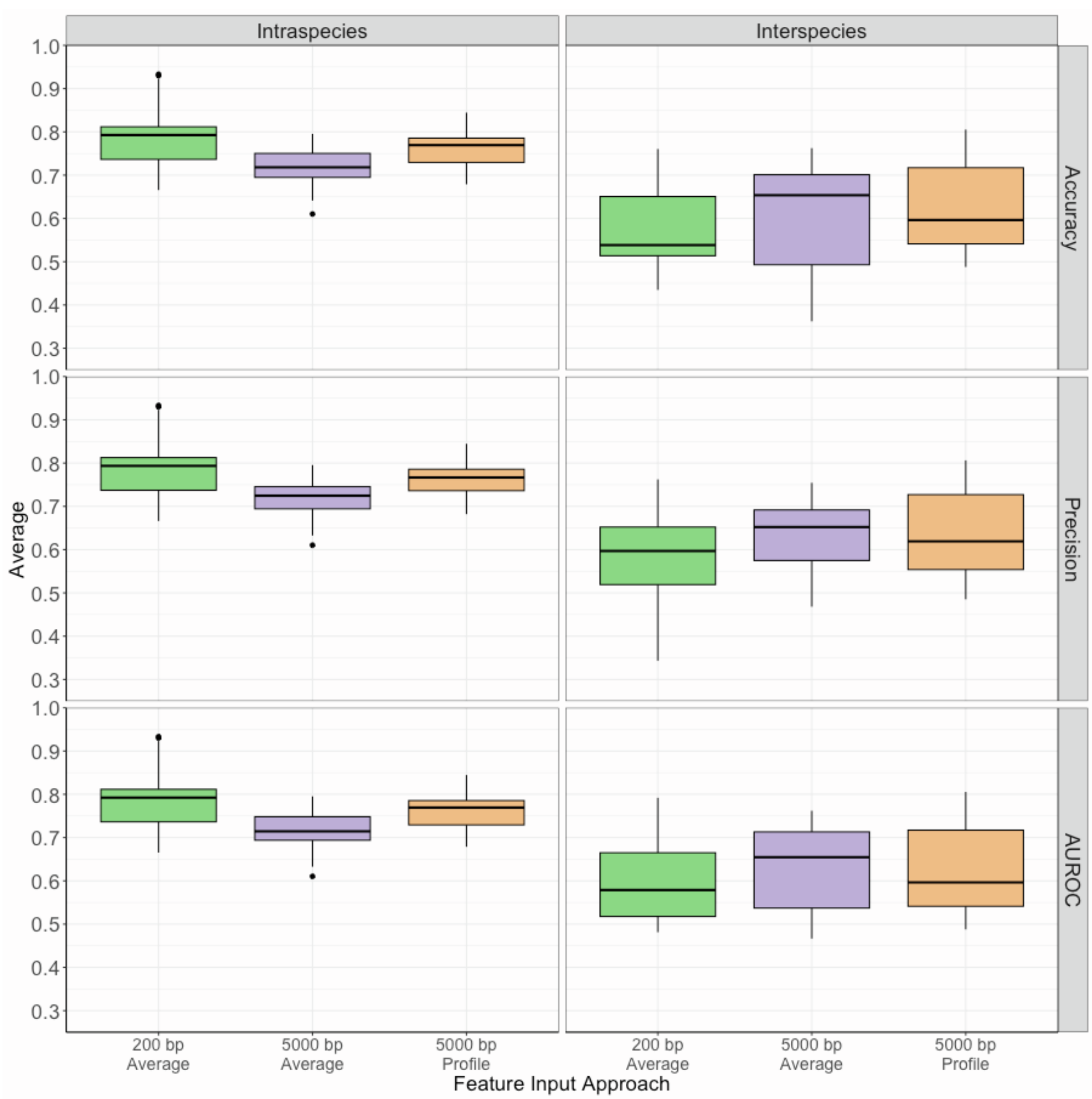

Supplementary Figure 6. Data pre-processing pipelines (related to Figures 5, 6 and 7).

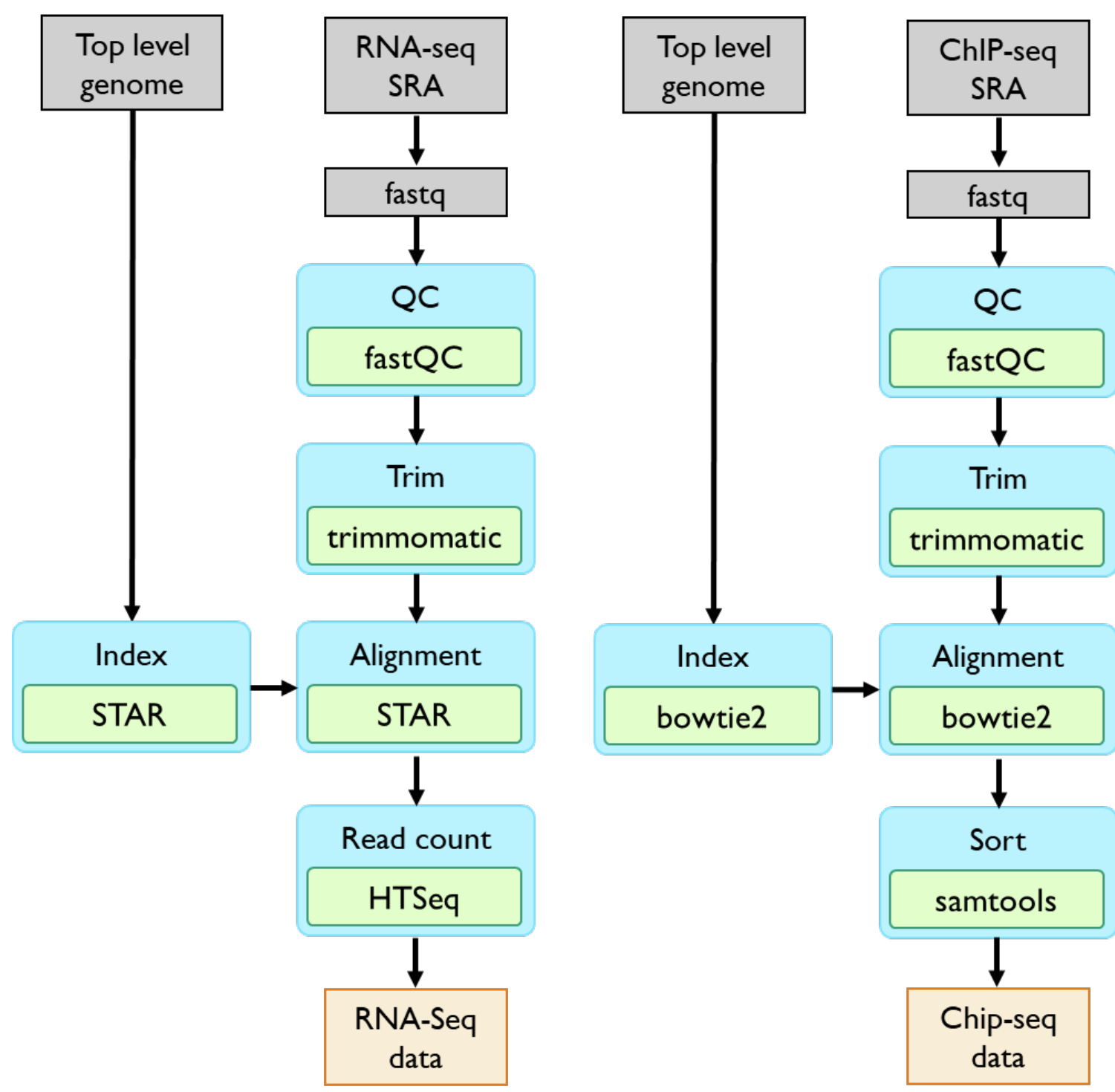

Supplementary Figure 7. Feature design strategy (related to Figures 5, 6 and 7).

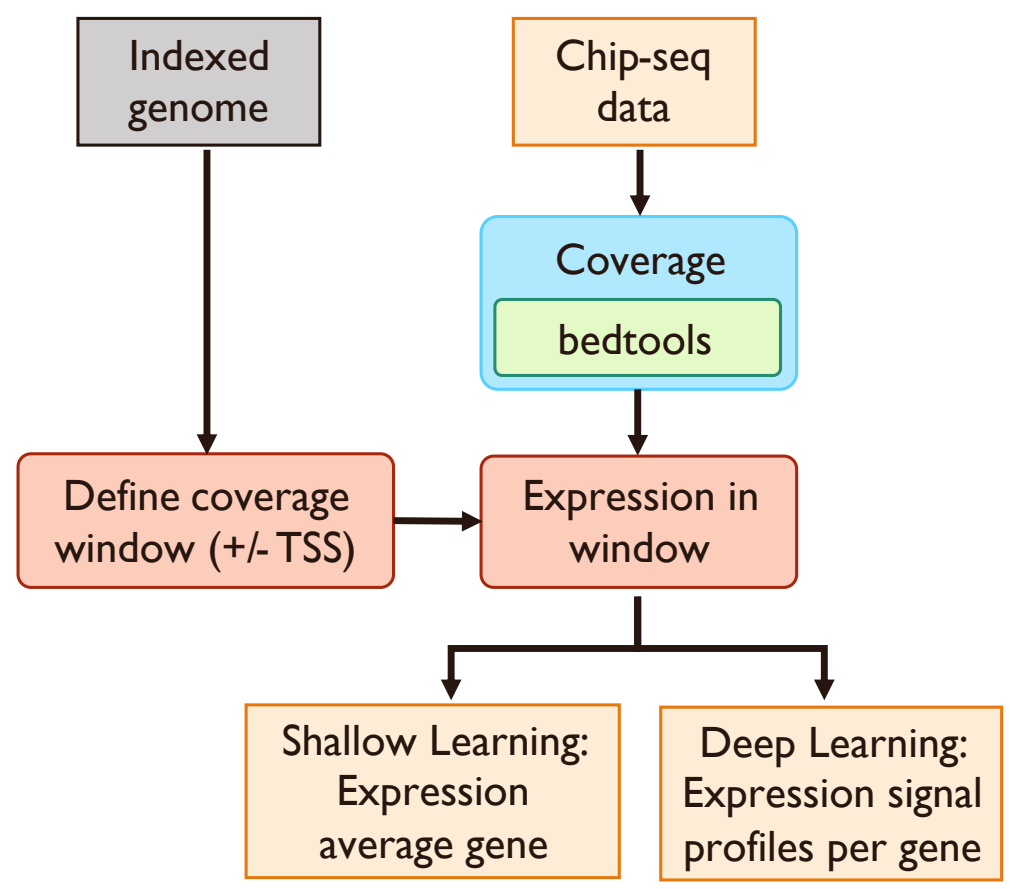

Supplementary Figure 8. Machine learning training and evaluation strategy (related to Figures 5, 6 and 7).

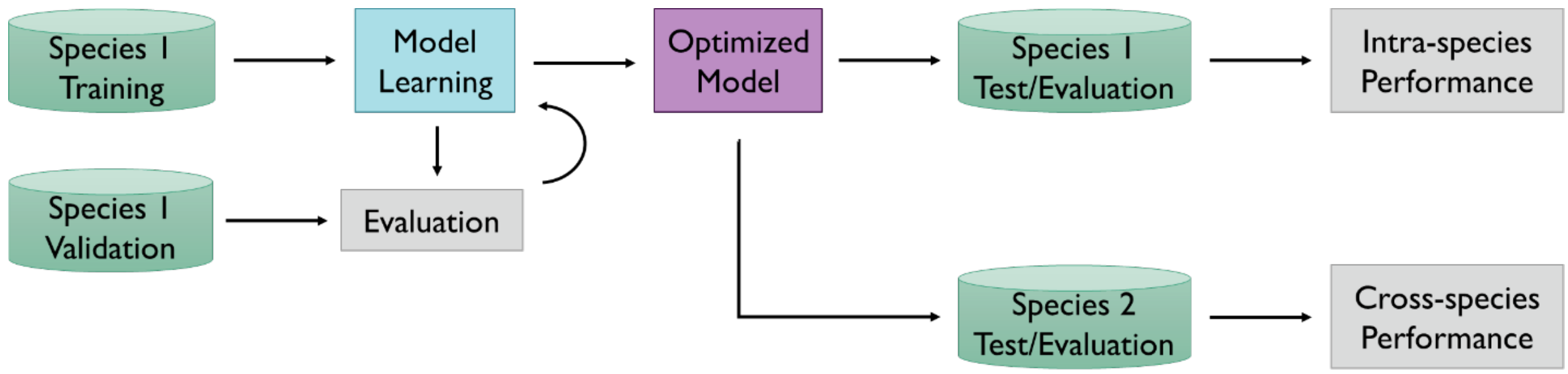

Supplementary Figure 9. Intra- and inter-species prediction task performance of the sequence-only 7B parameter Evo2 model using a 5kb TSS window on accuracy (left), precision (center), and area under the receiver operating curve (AUC; right) metrics. Boxes represent median +/- quartile (related to Figure 7).

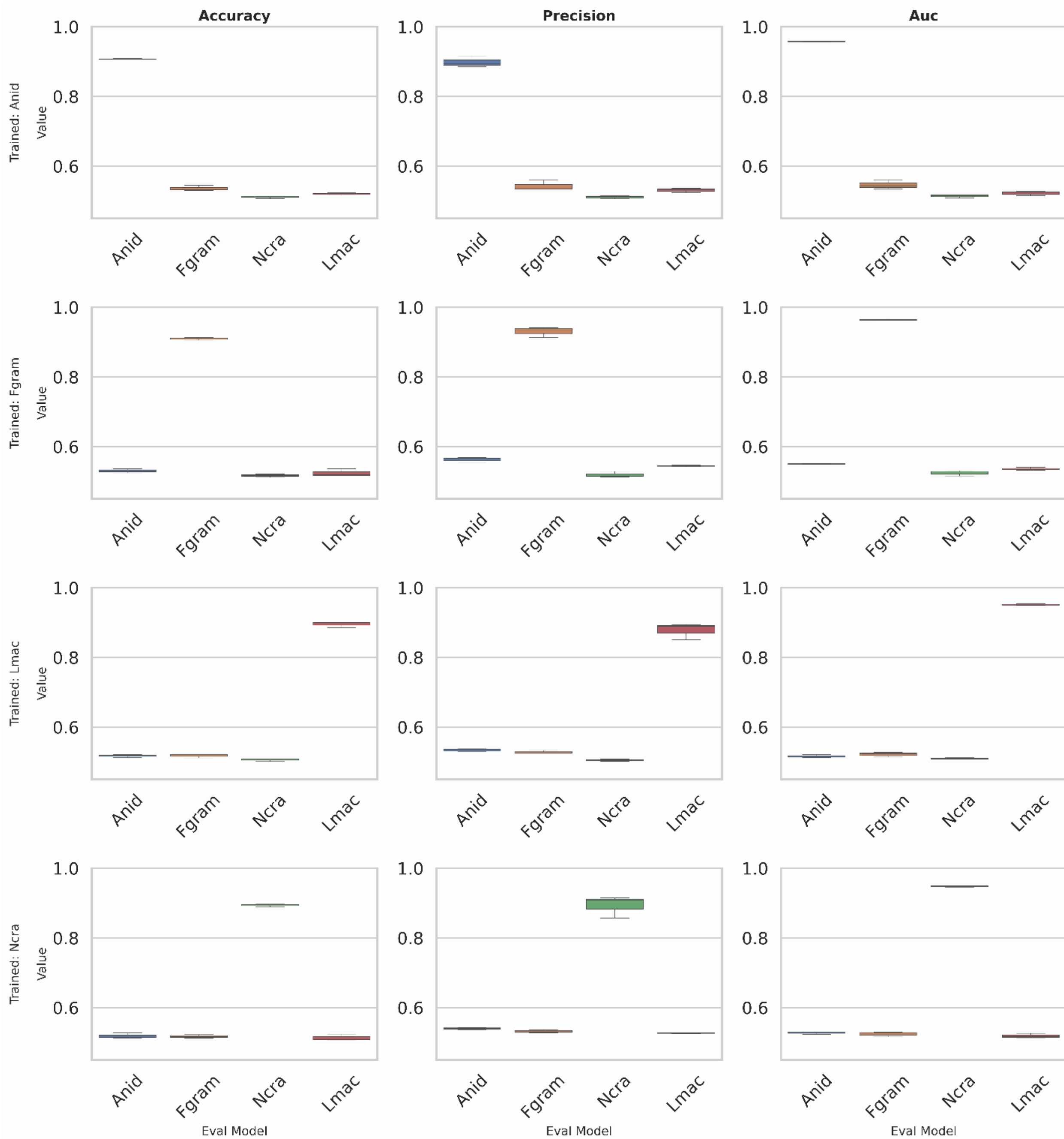

Supplementary Figure 10. Intra- and inter-species prediction task performance of the sequence-only 7B parameter Evo2 model using a 200bp TSS window on accuracy (left), precision (center), and area under the receiver operating curve (AUC; right) metrics. Boxes represent median +/- quartile (related to Figure 7).

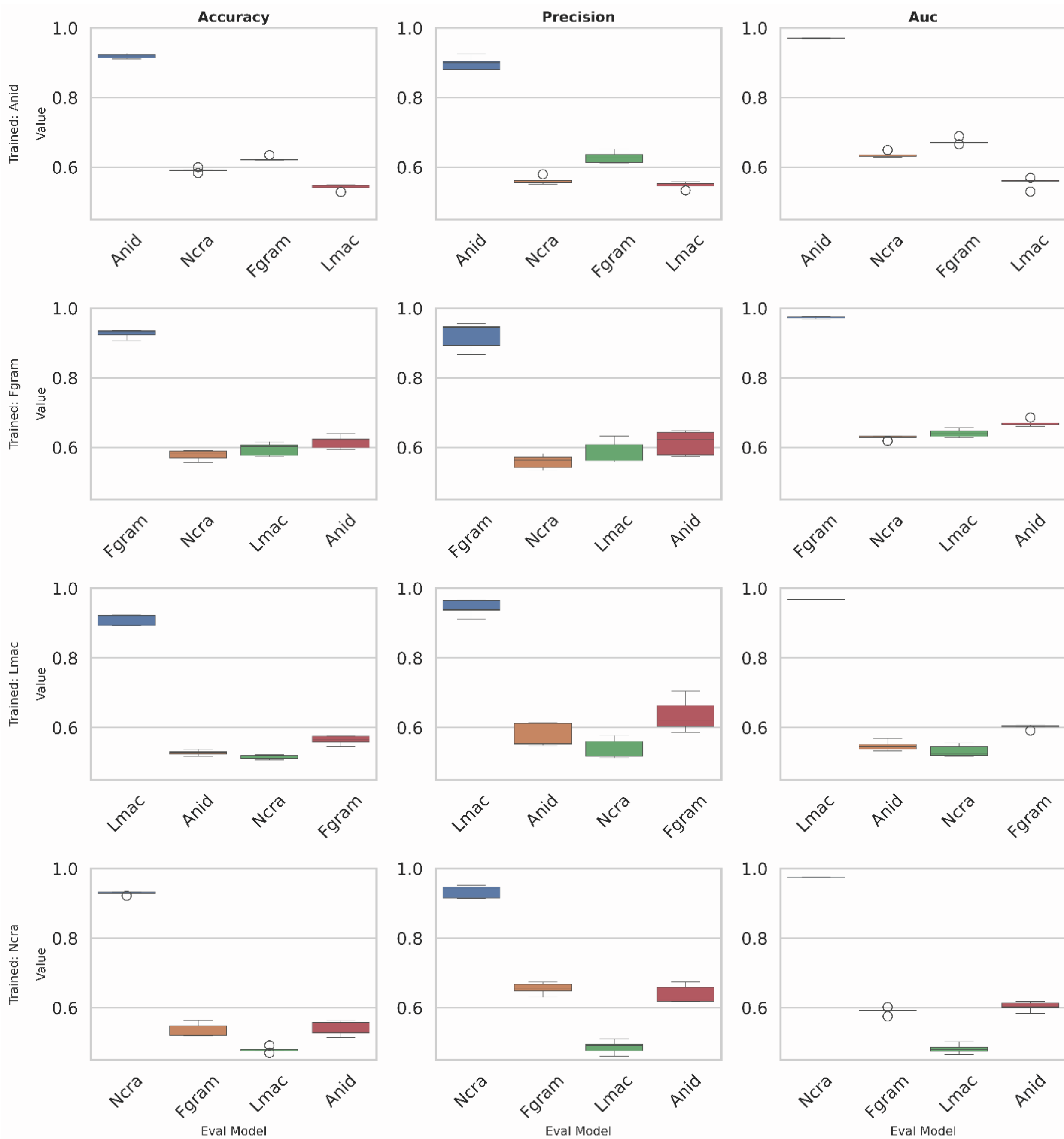

Supplementary Table 1. MAPLE vs. benchmark model intra- and inter-species prediction specificity (true negative rate) and sensitivity (true positive rate) (related to Figures 6 and 7).

Model	Trained	Tested	Specificity Mean	Specificity Std	Sensitivity Mean	Sensitivity Std
Benchmark	Anid	Anid	0.791	0.006	0.866	0.002
		Fgram	0.479	0.019	0.899	0.006
		Lmac	--	--	--	--
		Ncrassa	1	0	0.001	0.001
	Fgram	Anid	0.786	0.002	0.801	0.001
		Fgram	0.866	0.005	0.762	0.003
		Lmac	0.499	0.013	0.95	0.004
		Ncrassa	0.975	0.03	0.178	0.182
	Lmac	Anid	--	--	--	--
		Fgram	0.961	0	0.492	0.005
		Lmac	0.682	0.009	0.912	0.001
		Ncrassa	1	0	0	0
	Ncrassa	Anid	0.002	0	1	0
		Fgram	0.2	0.269	0.969	0.046
		Lmac	0.091	0.036	0.998	0
		Ncrassa	0.84	0.01	0.706	0.081
MAPLE	Anid	Anid	0.691	0.042	0.723	0.072
		Fgram	0.809	0.006	0.476	0.008
		Ncrassa	0.091	0.004	0.938	0.004
	Fgram	Anid	0.627	0.005	0.755	0.002
		Fgram	0.824	0.012	0.746	0.017
		Lmac	0.618	0.007	0.812	0.003
		Ncrassa	0.727	0.006	0.793	0.006
	Lmac	Fgram	0.927	0.002	0.504	0.005
		Lmac	0.681	0.013	0.797	0.011
		Ncrassa	0.765	0.014	0.775	0.011
	Ncrassa	Anid	0.518	0.003	0.831	0.002
		Fgram	0.767	0.024	0.756	0.002
		Lmac	0.555	0.008	0.825	0.007
		Ncrassa	0.505	0.248	0.836	0.036

Supplementary Table 2. MAPLE intra-species prediction performance for *A. nidulans* using modification subsets and varying TSS windows. Missing values are denoted with '--' (related to Figures 6 and 7).

	**TSS window**				
	**40kb**	**40kb**	**5kb**	**5kb**	**5kb**
**Modification(s) included**	**H3K36me3**	**H3K36me3 H3K4me3 H3K9me3**	**H3K9me3**	**H3K36me3**	**H3K36me3 H3K4me3 H3K9me3**
**AUROC**	--	--	0.528	0.734	0.820
**Accuracy**	0.711	0.716	0.519	0.687	0.752
